# Regulation of integrin and extracellular matrix genes by HNRNPL is necessary for epidermal renewal

**DOI:** 10.1371/journal.pbio.3001378

**Published:** 2021-09-20

**Authors:** Jingting Li, Yifang Chen, Manisha Tiwari, Varun Bansal, George L. Sen

**Affiliations:** 1 Institute of Precision Medicine, The First Affiliated Hospital, Sun Yat-Sen University, Guangzhou, China; 2 Department of Dermatology, Department of Cellular and Molecular Medicine, UCSD Stem Cell Program, University of California San Diego, La Jolla, California, United States of America; IMBA, AUSTRIA

## Abstract

Stratified epithelia such as the epidermis require coordinated regulation of stem and progenitor cell proliferation, survival, and differentiation to maintain homeostasis. Integrin-mediated anchorage of the basal layer stem cells of the epidermis to the underlying dermis through extracellular matrix (ECM) proteins is crucial for this process. It is currently unknown how the expression of these integrins and ECM genes are regulated. Here, we show that the RNA-binding protein (RBP) heterogeneous nuclear ribonucleoprotein L (HNRNPL) binds to these genes on chromatin to promote their expression. HNRNPL recruits RNA polymerase II (Pol II) to integrin/ECM genes and is required for stabilizing Pol II transcription through those genes. In the absence of HNRNPL, the basal layer of the epidermis where the stem cells reside prematurely differentiates and detaches from the underlying dermis due to diminished integrin/ECM expression. Our results demonstrate a critical role for RBPs on chromatin to maintain stem and progenitor cell fate by dictating the expression of specific classes of genes.

## Abstract 2

Maintenance of the epidermis requires integrin-mediated anchorage of basal layer stem cells of the epidermis to the underlying dermis via extracellular matrix (ECM) proteins. This study shows that the RNA binding protein HNRNPL binds to the chromatin of genes encoding integrin and ECM proteins to promote their expression by stabilizing RNA polymerase II-mediated transcription. In the absence of HNRNPL, the basal layer of the epidermis prematurely differentiates and detaches.

## Introduction

Integrins are large, transmembrane spanning proteins that are essential for tissue organization, survival, homeostasis, differentiation, and signal transduction [1]. Integrins form heterodimers between 18 α and 8 β subunits that can form up to 24 different receptors [2,3]. Integrins are important for epithelial tissues such as the epidermis to communicate as well as attach to the extracellular matrix (ECM) [4]. The human epidermis acts as an essential barrier between the body and the outside environment. It is a stratified epithelium that requires a proper balance of cell proliferation and differentiation to maintain its homeostasis and integrity [5]. Stem and progenitor cells in the basal layer of the epidermis maintain a population of proliferative cells while also giving rise to differentiated progeny. Those cells entering the differentiation program undergo various stages of differentiation as they migrate outwards through the epidermis, where they will eventually form the protective outer layer known as the stratum corneum. In order for the basal layer stem and progenitor cells to stay undifferentiated, they must attach to the underlying dermis through hemidesmosomes. Hemidesmosomes are made of cell surface integrins (i.e., α6β4 and α3β1) that connect to laminins and collagens in the basement membrane [6]. Human genetic mutations in these proteins (integrins, laminins, and collagens) lead to debilitating and life-threatening skin blistering diseases known as epidermolysis bullosa, which is characterized by the detachment of the epidermis from the dermis [7–11]. β1 integrin activation blocks epidermal differentiation and promotes stem cell expansion [12]. In addition, integrins such as α5β1 are necessary for epithelial movement [13]. Despite the importance of integrins and ECM proteins such as laminins to epithelial integrity, stem cell fate decisions, migration, and tumorigenesis, it is currently unclear how these genes are regulated.

Potential regulators of integrin expression include RNA-binding proteins (RBPs). The human genome encodes approximately 1,500 RBPs that are involved in all aspects of RNA metabolism such as splicing, export, stability and translation [14]. While most studies have focused on RBPs function in regulating RNA, there is increasing evidence that they may have direct roles in transcription. This includes SRSF2, which has been shown to be part of the 7SK complex and is critical for transcription pause release [15]. A recent large-scale analysis of 45 RBPs in K562 cells and 58 RBPs in HepG2 cells showed that approximately 60% of these factors associated with chromatin [16]. Binding to chromatin occurred through 2 ways. First, RBPs can directly bind DNA or be part of complexes assembled at gene promoters. Second, RBPs can associate with chromatin indirectly through nascent RNA to control gene expression. Despite major efforts to understand RBPs and their potential function on chromatin, >95% of RBPs mechanism of action as well as tissue function remains uncharacterized [16]. We have recently shown that in human epidermis, the RBP heterogeneous nuclear ribonucleoprotein K (HNRNPK) is necessary for epidermal proliferation and prevention of premature differentiation through its impacts on both the RNA and DNA level [17]. HNRNPK degrades the mRNAs of genes that code for differentiation promoting transcription factors such as GRHL3 and KLF4 in progenitor cells to prevent premature differentiation [17,18]. HNRNPK also binds to chromatin and is necessary to recruit RNA polymerase II (Pol II) to proliferation genes to promote epidermal growth. Because of HNRNPK’s importance to epidermal growth and differentiation, we hypothesized that other related RBPs might also be critical to epidermal function.

Through targeting candidate RBPs, we found that heterogeneous nuclear ribonucleoprotein L (HNRNPL) is necessary to maintain epidermal stem and progenitor cell identity. HNRNPL does this by binding to hemidesmosome and ECM genes and promoting their transcription by stabilizing Pol II binding to those genes. HNRNPL-mediated expression of those genes allows attachment of basal layer stem and progenitor cells to the underlying dermis, which prevents premature differentiation and anoikis. Our results have identified an RBP acting on chromatin to regulate integrin and ECM expression, which, in turn, maintains the stem and progenitor cell compartment of the epidermis.

## Results

### HNRNPL is necessary to prevent apoptosis and premature differentiation

**Fig 1:**
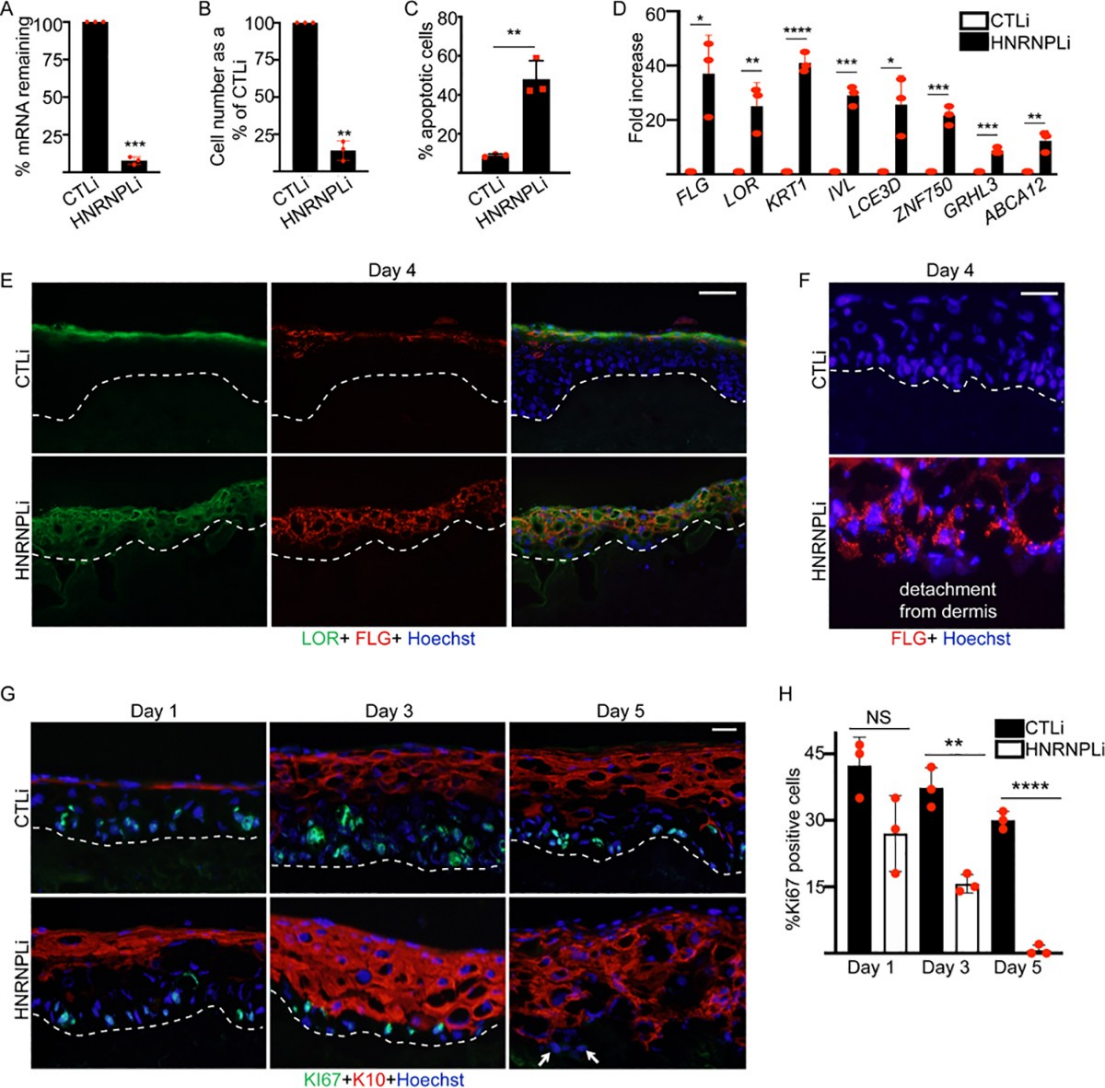
HNRNPL is required for maintaining epidermal progenitor cell function. **(A)** Primary human keratinocytes were knocked down with control (CTLi) or HNRNPL (HNRNPLi) siRNAs, and the remaining *HNRNPL* mRNA levels were measured using RT-qPCR. qPCR results were normalized to *L32* levels. **(B)** CTLi and HNRNPLi cells were seeded at 150,000 cells and counted 4 days later. Cell number was reported as a percentage of CTLi levels. **(C)** Annexin V staining was used to determine the percentage of apoptotic cells in CTLi and HNRNPLi cells after 4 days of culture. **(D)** RT-qPCR for epidermal differentiation gene expression in CTLi and HNRNPLi cells after 4 days of knockdown. qPCR results were normalized to *L32* levels. **(E)** Regenerated human skin using three-dimensional organotypic cultures made from CTLi or HNRNPLi cells were harvested after 4 days of culture. Immunostaining of late differentiation proteins LOR (green) and FLG (red) are shown. Nuclei are shown in blue (Hoechst staining). The dashed white lines denote the basement membrane zone. Scale bar = 40 μm. **(F)** High magnification images of day 4 regenerated human skin in CTLi and HNRNPLi samples. Staining of FLG (red) merged with nuclei (Hoechst) is shown. Scale bar = 10 μm. **(G)** Time course (days 1, 3, and 5) of human skin regeneration. Immunostaining of CTLi or HNRNPLi regenerated human skin with early differentiation marker K10 (red) and proliferation marker KI67 (green). White arrowheads denote rare basal layer cells not stained with K10 in HNRNPLi samples. Scale bar = 10μm. **(H)** Quantification of the percentage of Ki67 positive cells in the basal layer from (G). *N* = 3 independent experiments for all of Fig 1. Mean values are shown with error bars = SD. *p < 0.05, ** ***p*** < 0.01, ***p < 0.001, ****p < 0.0001 (*t* test). NS, not significant. Primary data for this figure can be found in S1 Data. FLG, filaggrin; HNRNPL, heterogeneous nuclear ribonucleoprotein L; K10, keratin 10; LOR, loricrin; RT-qPCR, reverse transcription quantitative PCR; siRNA, small interfering RNA.

To determine whether other heterogeneous nuclear ribonucleoproteins (HNRNPs) are important for epidermal growth and differentiation, we targeted several high-expressing HNRNPs (based on our previous RNA sequencing [RNA-seq] results [17,19]) using small interfering RNAs (siRNAs) against HNRNPL, HNRNPF, HNRNPH1, and HNRNPH2 in primary human keratinocytes. Depletion of HNRNPF, HNRNPH1, and HNRNPH2 greater than 80% had no impacts on epidermal proliferation or expression of differentiation genes such as *IVL* or *FLG* (S1A–S1D Fig). In contrast, loss of HNRNPL severely decreased cell numbers partly due to a large increase in cells undergoing apoptosis (Fig 1A–1C). HNRNPL knockdown cells also started expressing differentiation genes such as *FLG*, *LOR*, *KRT1*, *IVL*, *LCE3D*, *ZNF750*, *GRHL3*, and *ABCA12* despite the cells being grown in proliferation conditions (Fig 1D). These differentiation genes are critical to epidermal structure and function. Mutations in these genes lead to human genetic skin diseases such as epidermolytic hyperkeratosis (KRT1), ichthyosis vulgaris (FLG), seborrheic dermatitis (ZNF750), psoriasis (LCE3D), and harlequin ichthyosis (ABCA12) [20–24]. We also used retroviral delivery of short hairpin RNAs (shRNAs) to knockdown HNRNPL to complement our siRNA results. Depletion of HNRNPL using 2 distinct shRNAs (shRNAs A and B) targeting different regions of HNRNPL resulted in loss of cell number as well as up-regulation of epidermal differentiation genes (S1E–S1G Fig). To determine the impacts of HNRNPL loss in a tissue setting that allows cell–cell and cell basement membrane contact in a three-dimensional context, control (CTLi) and HNRNPL knockdown (HNRNPLi) primary human keratinocytes were seeded onto devitalized human dermis to regenerate human skin [17,18,25–27]. In control tissue, late terminal differentiation proteins filaggrin (FLG) and loricrin (LOR) were properly expressed and localized to the outermost layer of the epidermis (stratum corneum) (Fig 1E). Interestingly, loss of HNRNPL caused FLG and LOR to be expressed throughout the entire epidermis even in the basal layer where the stem and progenitor cells reside (Fig 1E and 1F). Apoptosis as assayed by the presence of cleaved caspase 3 was found throughout the HNRNPLi epidermis (S2A Fig). Large clumps of apoptotic cells were also detected at the top of the epidermis in HNRNPLi tissue (S2A Fig, white arrows). To understand the kinetics of HNRNPL’s role on epidermal growth and differentiation, a time course of human skin regeneration was performed. After only 1 day of seeding the control and HNRNPL knockdown keratinocytes onto human dermis, the HNRNPLi tissue already had robust expression of early differentiation protein keratin 10 (K10) (Fig 1G). This was in contrast to control tissue where K10 was just starting to be expressed (Fig 1G). The basal layer of the HNRNPLi epidermis became progressively more differentiated, and, by day 5, there were few cells not expressing K10 (Fig 1G). This premature differentiation coincided with a significant loss of proliferative, Ki67 positive cells in the basal layer (Fig 1G and 1H). In human skin, HNRNPL was expressed in the nucleus in all layers of the epidermis (S2B Fig). Similarly, HNRNPL protein levels did not change upon induction of epidermal differentiation (S2C Fig). These results suggest that HNRNPL is required to prevent premature differentiation and apoptosis while promoting self-renewal of the epidermis.

### HNRNPL promotes expression of hemidesmosome and extracellular matrix genes while suppressing differentiation genes

**Fig 2:**
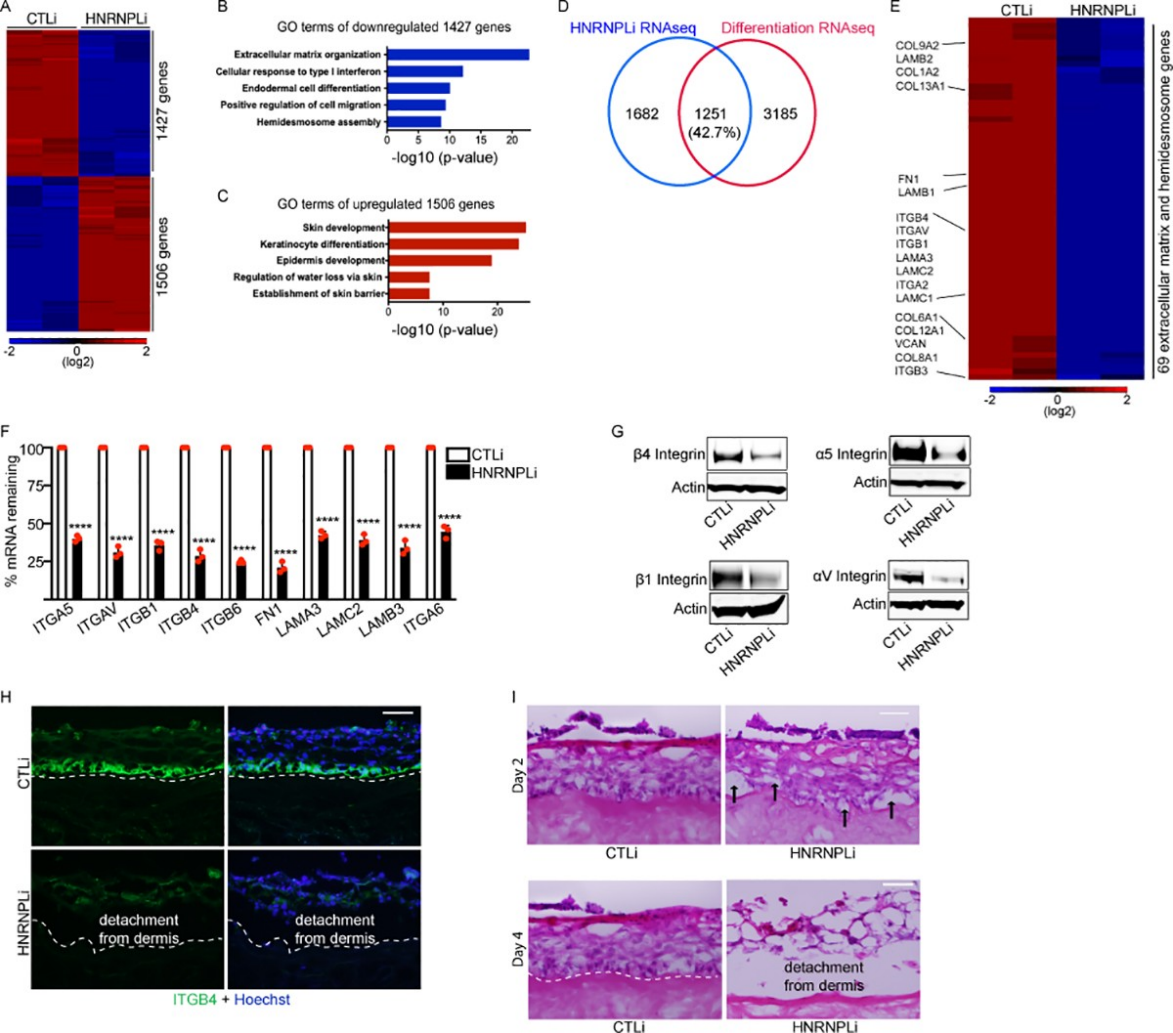
HNRNPL promotes the expression of integrin and ECM genes to allow attachment of the epidermis to dermis. **(A)** RNA-seq analysis of CTL and HNRNPL knockdown primary human keratinocytes (day 4). RNA-seq results were performed in duplicates. A total of 1,506 genes were up-regulated (red), and 1,427 genes were down-regulated (blue) upon HNRNPL depletion. Heatmap is shown in log2 scale. **(B)** GO terms for the 1,427 genes down-regulated in HNRNPLi cells. **(C)** GO terms for the 1,506 genes with increased expression upon HNRNPL loss. **(D)** Overlap of the HNRNPL knockdown gene expression signature with the differentiation signature. **(E)** Heatmap showing the 69 ECM and hemidesmosome genes down-regulated upon HNRNPL knockdown. Heatmap is shown in log2 scale. **(F)** RT-qPCR for integrin and ECM gene expression in CTLi and HNRNPLi cells after 4 days of knockdown. qPCR results were normalized to *L32* levels. Mean values are shown with error bars = SD and ****p < 0.0001 (*t* test). **(G)** Western blot for α and β integrin protein levels in CTLi and HNRNPLi cells. Representative images are shown. **(H)** Immunostaining of CTLi or HNRNPLi regenerated human skin (day 4) with β4 integrin (ITGB4: green). Nuclei are shown in blue (Hoechst staining). The dashed white lines denote the basement membrane zone. Scale bar = 40 μm. **(I)** Time course (days 2 and 4) of human skin regeneration. Hematoxylin–eosin staining of regenerated CTLi and HNRNPLi human skin. Black arrowheads denote areas of epidermal detachment from the dermis. Scale bar = 40 μm. *N* = 3 independent experiments for Fig 2 unless otherwise specified. Primary data for this figure can be found in S1 Data. ECM, extracellular matrix; GO, gene ontology; HNRNPL, heterogeneous nuclear ribonucleoprotein L; RNA-seq, RNA sequencing; RT-qPCR, reverse transcription quantitative PCR.

To determine the genes that HNRNPL is regulating, we performed RNA-seq on CTLi and HNRNPLi primary human keratinocytes. A total of 1,427 genes (*p*-value < 0.05 and ≥2-fold change) decreased in expression upon HNRNPL knockdown, which were enriched in genes involved in ECM organization and hemidesmosome assembly (Fig 2A and 2B, S1 Table). Moreover, 1,506 genes were up-regulated upon HNRNPL depletion, which were enriched in gene ontology (GO) terms such as skin development and keratinocyte differentiation (Fig 2A and 2C, S1 Table). A comparison with the differentiation gene expression signature (genes that changed upon epidermal differentiation obtained from a previous publication [28]) showed that close to 43% of the HNRNPL gene signature overlapped (Fig 2D). This suggests that HNRNPL is critical to prevent premature unraveling of the epidermal differentiation gene expression program. Notably, 2 critical gene classes were down-regulated in HNRNPLi cells including ECM organization and hemidesmosome assembly genes (Fig 2E, S2 Table). This comprised of 69 genes including collagens, integrins, and laminins (Fig 2E, S2 Table). Attachment of the basal layer of the epidermis to the dermis depends on those proteins and without it; the cells would prematurely differentiate as well as result in anoikis [6,29–31]. Thus, down-regulation of these critical attachment genes could explain the large increase in apoptotic cells as well as the premature differentiation phenotype of HNRNPL-depleted cells. Validation of these results by reverse transcription quantitative PCR (RT-qPCR) showed that HNRNPL loss resulted in decreased expression of integrins (*ITGA5*, *ITGAV*, *ITGA6*, *ITGB1*, *ITGB4*, and *ITGB6*), laminins (*LAMA3*, *LAMC2*, and *LAMB3*), and fibronectin1 (*FN1*) (Fig 2F). Importantly, protein levels of β4 (ITGB4), α5 (ITGA5), β1 (ITGB1), and αV (ITGAV) integrins were also diminished upon HNRNPL loss (Fig 2G). In tissue, HNRNPL knockdown resulted in loss of β4 integrin (ITGB4), which is necessary for the attachment to the underlying dermis (Fig 2H). Two days after seeding HNRNPLi keratinocytes onto dermis, there were local areas of detachment that progressed to complete detachment by day 4 (Fig 2I). This loss of epidermal attachment to the underlying dermis is similar to the clinical phenotypes seen in epidermolysis bullosa patients.

### HNRNPL binds to hemidesmosome and cell-substrate junction assembly genes and promotes their expression

**Fig 3:**
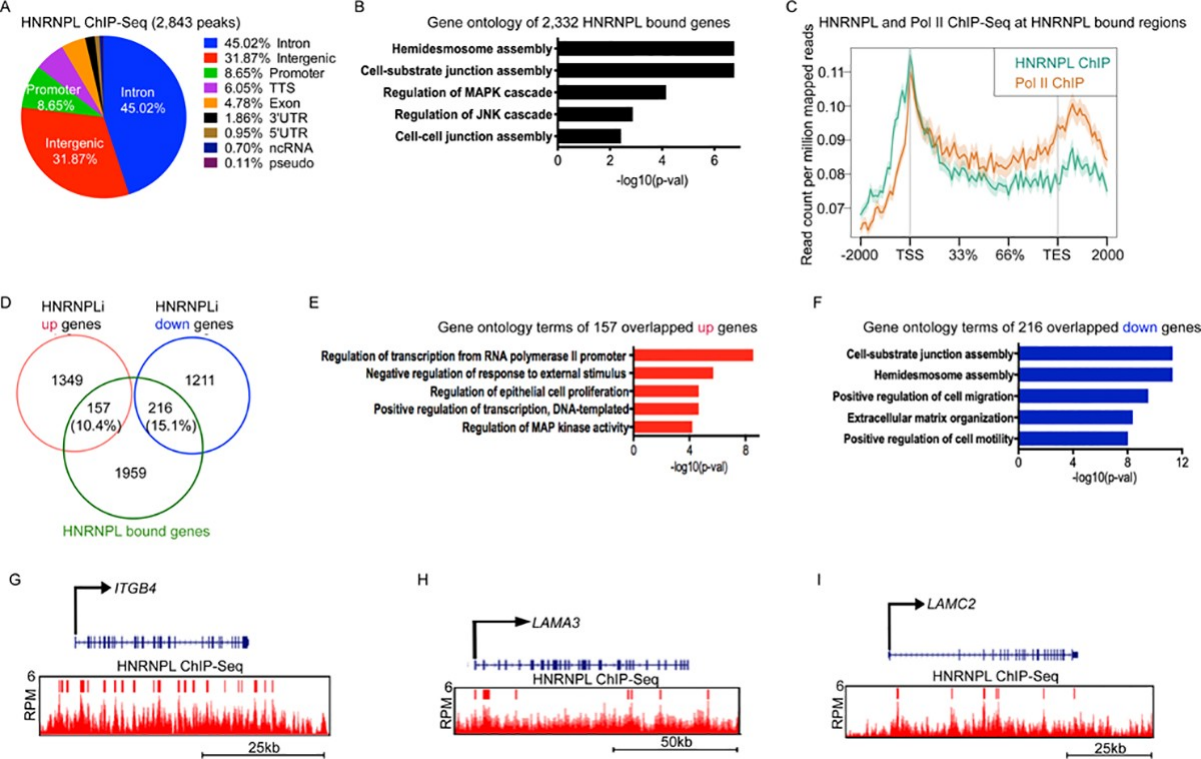
HNRNPL binds to hemidesmosome and cell-substrate assembly genes. **(A)** Genomic localization of the 2,843 HNRNPL bound peaks. The percent of HNRNPL binding to each genomic region is shown. HNRNPL ChIP-Seq was performed in primary human keratinocytes in growth conditions (*n* = 3 independent experiments). **(B)** GO terms of the 2,332 genes that the 2,843 HNRNPL bound peaks mapped to. **(C)** Metagene plot of HNRNPL (teal) and RNA Pol II (orange) ChIP-Seq reads at HNRNPL bound regions. Y-axis is shown as read count per million reads, and X-axis is the distance along HNRNPL bound genes. TSS is transcription start site, and TES is transcription end site. **(D)** Venn diagram of HNRNPL bound genes (HNRNPL ChIP-Seq) with genes up-regulated or down-regulated upon HNRNPL depletion. **(E)** GO terms of the HNRNPL bound genes that overlap with genes increased upon HNRNPL loss. **(F)** GO terms of the 216 decreased genes upon HNRNPL knockdown that overlap with HNRNPL bound genes. **(G–I)** Gene tracks of *ITGB4* (G), *LAMA3* (H), and *LAMC2* (I). HNRNPL ChIP-Seq is shown in red. Y-axis shows RPM, and red bar over gene tracks represent significant peaks. X-axis shows position along gene. Primary data for this figure can be found in S1 Data. ChIP-Seq, chromatin immunoprecipitation sequencing; GO, gene ontology; HNRNPL, heterogeneous nuclear ribonucleoprotein L; Pol II, polymerase II; RPM, reads per million.

Since HNRNPL was one of the RBPs found to bind chromatin in a large-scale analysis [16] of 45 RBPs in K562 cells and 58 RBPs in HepG2 cells, we performed chromatin immunoprecipitation sequencing (ChIP-Seq) on HNRNPL in proliferating primary human keratinocytes to determine the genes that it may be directly regulating. HNRNPL bound to 2,843 peaks, with approximately 68% of the bound sites mapping back to genic regions (5′ UTR, promoter, intron, exon, TTS, 3′ UTR) (Fig 3A). The 2,843 peaks mapped back to 2,332 genes that were enriched for GO terms such as hemidesmosome assembly, cell-substrate junction assembly, regulation of mitogen-activated protein kinase (MAPK) cascade, and cell–cell junction assembly (Fig 3B, S3 Table). A de novo motif search of the peaks that HNRNPL bound using HOMER [32] showed enrichment for transcription factors such as RUNX2, TWIST2, FOXH1, and SOX9 (S3A Fig). Of import, RUNX2 and SOX9 have been shown to be necessary for skin self-renewal and growth, suggesting that HNRNPL may be cooperating with these factors to maintain the stem cell state [33–35]. Since the vast majority of HNRNPL binding occurred within genic regions, we visualized how HNRNPL bound to these regions. HNRNPL binding peaked at the transcription start site (TSS) and was bound throughout the genic regions with a second peak near the transcription end site (TES) (Fig 3C). This binding was reminiscent of the RNA Pol II binding we had previously published [17,19,36] in human keratinocytes, suggesting that HNRNPL may be regulating transcription (Fig 3C). Moreover, 10.4% of the genes up-regulated upon HNRNPL depletion were bound by HNRNPL, which were enriched in GO terms such as regulation of transcription (Fig 3D and 3E). A total of 216 (15.1%) of the genes down-regulated upon HNRNPL loss were also bound by HNRNPL and were enriched for cell-substrate assembly, hemidesmosome assembly, and ECM organization genes (Fig 3D and 3F). This included genes such as *ITGB4*, *LAMA3*, and *LAMC2*, which are critical for the attachment of basal layer stem and progenitor cells to the underlying dermis (Fig 3G and 3I). A zoomed out view of the *ITGB4* genomic region with its proximal genes *UNK* and *SAP30BP* also confirmed the specificity of HNRNPL binding to *ITGB4* (S3B Fig). To test the specificity of HNRNPL binding to integrin/ECM genes, we performed ChIP-qPCR to determine if HNRNPL knockdown could reduce the binding to those regions. ChIP-qPCR showed that HNRNPL bound to *ITGA3*, *ITGB1*, *LAMB3*, and *ITGB4* in control cells, but the binding was significantly diminished upon HNRNPL knockdown (S3C Fig). HNRNPL did not bind to genes such as *TP63*, *DHFR*, and *PGK1*, which demonstrates its preference toward integrin/ECM genes (S3C Fig). These results suggest that HNRNPL directly regulates hemidesmosome/ECM genes and indirectly regulates differentiation through attachment of the basal layer to the underlying dermis.

### HNRNPL is necessary for Pol II binding and transcription of hemidesmosome and cell-substrate junction assembly genes

**Fig 4:**
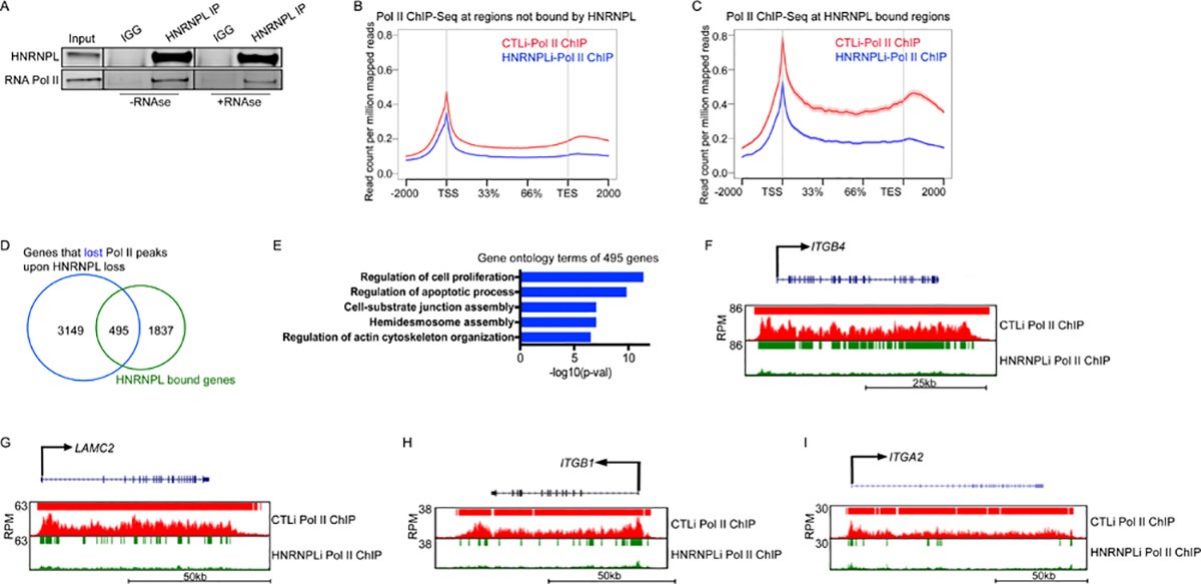
HNRNPL is required for Pol II–mediated transcription of integrin/ECM genes. **(A)** IPs were performed using an HNRNPL antibody or IGG and western blotted for HNRNPL or RNA Pol II protein expression. IPs were performed +/− RNase A. A total of 1% of the cell lysate was used as input. Representative blots are shown. **(B)** RNA Pol II ChIP-Seq at regions not bound by HNRNPL in CTLi (red) and HNRNPLi (blue) cells. Y-axis is shown as read count per million reads, and X-axis is the distance along genes. TSS is transcription start site, and TES is transcription end site. Pol II ChIP-Seq in CTLi and HNRNPLi cells were performed in duplicates. **(C)** Pol II ChIP-Seq at regions bound by HNRNPL in CTLi (red) and HNRNPLi (blue) cells. **(D)** Overlap between HNRNPL bound genes (HNRNPL ChIP-Seq) and genes that lost Pol II peaks upon HNRNPL knockdown. **(E)** GO terms of the 495 genes that overlap from (D). **(F–I)** Gene tracks of *ITGB4* (F), *LAMC2* (G), *ITGB1* (H), and *ITGA2* (I). Pol II ChIP-Seq is shown in CTLi (red) and HNRNPLi (green) cells. Y-axis shows RPM, and red or green bar over gene tracks represents significant peaks. X-axis shows position along gene. Primary data for this figure can be found in S1 Data. ChIP-Seq, chromatin immunoprecipitation sequencing; ECM, extracellular matrix; GO, gene ontology; HNRNPL, heterogeneous nuclear ribonucleoprotein L; IGG, immunoglobulin; IP, immunoprecipitation; Pol II, polymerase II; RPM, reads per million.

Because HNRNPL’s binding on chromatin resembled Pol II, we wanted to determine if HNRNPL associated with Pol II. Immunoprecipitation (IP) of HNRNPL showed that it could associate with Pol II even in the absence of RNA (Fig 4A). These results suggest that HNRNPL may potentially be regulating transcription of hemidesmosome and ECM genes in 2 main ways: (1) HNRNPL may be promoting the transcription elongation of HNRNPL bound genes. This is a possibility since HNRNPL has been previously found to interact with transcription elongation factors such as P-TEFb [37]. If this were the case, then HNRNPL knockdown should result in Pol II accumulating at the TSS/promoter regions. This would be accompanied by a loss of Pol II from the gene body regions of HNRNPL bound genes. (2) HNRNPL may be necessary for promoting the stability of Pol II binding along the TSS and gene body. If this were true, then HNRNPL depletion should result in Pol II loss from both the gene body and TSS of HNRNPL bound genes. To test these possibilities, we performed Pol II ChIP-Seq on control (CTLi) and HNRNPL knockdown (HNRNPLi) cells (S4A Fig). On genes not bound by HNRNPL, there was a slight loss of Pol II binding from the gene body and TSS regions in HNRNPL-depleted cells (Fig 4B). However, on genes bound by HNRNPL, there was a dramatic reduction in Pol II binding along the gene body and TSS regions in HNRNPL knockdown cells (Fig 4C). Approximately 21% (495/2,332) of HNRNPL bound genes lost Pol II peaks upon HNRNPL knockdown (Fig 4D, S4 Table). These 495 genes were enriched for GO terms such as cell-substrate junction and hemidesmosome assembly genes (Fig 4E, S4 Table). Notably, 51% (111/216) of the HNRNPL bound genes that are down-regulated upon HNRNPL knockdown also lost Pol II peaks (S4B Fig). These 111 genes were also highly enriched for GO terms such as ECM organization, cell-substrate junction assembly, and hemidesmosome assembly (S4C Fig). These HNRNPL bound genes include *ITGB4*, *LAMC2*, *ITGB1*, *ITGA3*, *ITGA2*, *LAMA3*, and *FN1* where HNRNPL loss led to depletion of Pol II from both the gene body and TSS (Fig 4F–4I, S4D–S4F Fig). Validation of these results by ChIP-qPCR also showed that HNRNPL knockdown led to a significant loss of Pol II binding to the TSS of *ITGB4*, *ITGB1*, *ITGA3*, *FN1*, and *LAMB3* (S4G Fig). Since HNRNPL could potentially act as a repressor at its bound genes, we overlapped the genes that gained Pol II peaks upon HNRNPL knockdown with its bound genes. A total of 430 of those genes overlapped, which were enriched for GO terms such as peptidyl-tyrosine phosphorylation and positive regulation of transcription (S4H and S4I Fig). While it is possible that HNRNPL is acting as a repressor at those genes, it is unlikely that those genes are a major contributor to the observed phenotype. Altogether, our data suggest that HNRNPL is necessary to promote the stability of Pol II transcription through hemidesmosome and ECM genes.

## Discussion

HNRNPL has been shown to be involved in most aspects of RNA regulation including RNA stability, splicing, as well as association with long noncoding RNAs (lncRNAs) to control gene expression [37–40]. It has been mostly studied in the context of cancer where it is required for tumor growth and metastasis [41]. In this context, HNRNPL interacts with various lncRNAs (specific lncRNAs dependent on tumor type) to promote tumorigenesis [42–46]. For example, the complex of lncRNA, DSCAM-AS1, and HNRNPL are necessary for breast cancer progression and tamoxifen resistance [42]. In hepatocellular carcinoma, the lncRNA SPRY4-IT1 associates with HNRNPL to promote growth and metastasis through the tumor necrosis factor (TNF) pathway [43]. Intriguingly, very little is known about the role of HNRNPL during normal tissue homeostasis as well as how it impacts gene expression by binding chromatin. In a large-scale study, HNRNPL was found to bind chromatin, but not much characterization was done on how it impacted gene expression [16].

Our results suggest HNRNPL is necessary to sustain epidermal stem and progenitor cells in the basal layer of the epidermis. In its absence, primary human keratinocytes undergo apoptosis, stop proliferating, and prematurely differentiate. In regenerated human skin, HNRNPL loss leads to terminal differentiation proteins being expressed in the basal layer of the epidermis, suggesting that the stem cell compartment has been lost. Apoptosis could be observed throughout the epidermis. In addition, the epidermis could no longer attach to the underlying dermis due to loss of expression of hemidesmosome/ECM genes. This phenotype resembled what is seen in epidermolysis bullosa patients who have mutations in integrin, laminin, or collagen. Integrin and ECM genes has been extensively studied; however, there is limited understanding on how these crucial genes are regulated on the transcriptional level [47,48]. Surprisingly, our HNRNPL ChIP-Seq data showed that HNRNPL bound genes were significantly enriched for hemidesmosome and cell-substrate junction assembly GO terms. This includes genes such as *ITGB4* and *LAMC2* that are crucial for the attachment of the basal layer to the dermis. It should be noted that HNRNPL binding to chromatin is weaker than other factors that have been traditionally characterized such as transcription or epigenetic factors. This weaker association was also observed by Xiao and colleagues when they mapped the DNA binding of a large number of RBPs [16]. Interestingly, HNRNPL binding to chromatin resembled that of Pol II and the 2 proteins also associate. This suggests that HNRNPL may be crucial for Pol II–mediated transcription on HNRNPL bound genes. Supporting this, knockdown of HNRNPL substantially decreased Pol II binding across HNRNPL bound genes that were enriched for hemidesmosome and ECM genes. Importantly, Pol II was only minimally impacted on genes not bound by HNRNPL.

Our data suggest that HNRNPL indirectly regulates differentiation and apoptosis since HNRNPL did not bind to these gene classes. Genes up-regulated (or gained Pol II peaks) upon HNRNPL depletion and were bound by HNRNPL were not enriched for differentiation or apoptosis genes. Instead, these genes were involved in the regulation of transcription from RNA Pol II promoter. Thus, HNRNPL indirectly prevents premature differentiation and anoikis by promoting the expression of hemidesmosome/ECM genes. This, in turn, allows attachment of the basal layer to the underlying dermis [6,49,50]. Previous studies have shown that keratinocytes grown in suspension without anchorage to the ECM spontaneously differentiate as it is mimicking the process of delamination from the basal layer [6,29,30]. A prior study showed that HNRNPL binds to muscle differentiation genes and activates their expression [51]. This is in contrast to our study where HNRNPL does not bind to differentiation genes in keratinocytes. HNRNPL is also necessary for maintaining the undifferentiated state in keratinocytes rather than promoting differentiation as shown in the muscle study. It is currently unclear whether the differences are due to cell type–specific differences (keratinocytes versus myoblasts) or the primary cells that were used in this study as compared to the transformed muscle cell line used in the previous study.

Since HNRNPL can bind to RNA, it is also possible that HNRNPL binds to RNA to control epidermal growth and differentiation. For example, HNRNPL could potentially control the mRNA stability or translation of skin master transcription factors such as *P63* to promote expression of hemidesmosome/ECM genes. However, the major phenotypes of premature differentiation, apoptosis, and detachment of the epidermis from the dermis can be explained through transcriptional control of hemidesmosome and ECM genes through HNRNPL. Our model suggests that lineage-specific transcription factors open the chromatin to hemidesmosome/ECM genes, which then allows HNRNPL to maintain Pol II transcription at those genes. This is supported by our findings that HNRNPL bound genes were enriched with SOX9 and RUNX2 binding motifs. With HNRNPL being expressed throughout the epidermis, basal layer–specific transcription factors such as SOX9 [34,35] could potentially recruit HNRNPL to target genes to achieve layer-specific gene expression. In summary, we have elucidated how HNRNPL maintains epidermal renewal by promoting the expression of adhesion genes through RNA Pol II, which then allows attachment of the epidermis to the dermis.

## Methods

### Cell culture

Primary human epidermal keratinocytes (derived from neonatal foreskin) were purchased from Life Technologies (Carlsbad, California, USA) (C0015C) and cultured in EpiLife medium (Life Technologies: MEPI500CA). This media was supplemented with penicillin/streptomycin (HyClone SV30010) and human keratinocyte growth supplement (Life Technologies: S0015). Cells were induced to differentiate in full confluence and 1.2 mM calcium for 3 and 5 days. Phoenix cells were cultured in Dulbecco’s Modified Eagle Medium (DMEM) with 10% fetal calf serum.

### Gene knockdown

siRNAs were placed into EpiLife media with the transfection reagent Lipofectamine RNAiMAX (25 ul for 10-cm plate transfection and 50 ul for 15-cm plate transfection) (Thermo Fisher Scientific (Waltham, MA): 13778) and incubated for 5 minutes at room temperature. This siRNA media was then diluted 1:10 and added to subconfluent keratinocytes, with the siRNA at a final concentration of 10 nM. The keratinocytes were then incubated in this media for a minimum of 18 hours to carry out siRNA knockdown. The siRNAs used in this study are as follows: Control siRNA (Ambion (Carlsbad, CA) Silencer Select Negative Control 4390844), HNRNPL siRNA (Ambion Silencer Select s6742), HNRNPF (Dharmacon (Lafayette, CO) M-013449-02-0005), HNRNPH1 siRNA (Dharmacon M-012107-00-0005), and HNRNPH2 siRNA (Dharmacon M-013245-01-0005). The retroviral constructs (3 ug) expressing shRNAs were transfected using Fugene 6 (Roche, Basel, Switzerland) into amphotrophic phoenix cells to knockdown genes. Viral supernatants were collected 48 hours posttransfection and used to infect primary human keratinocytes. Cells were incubated in the viral supernatants and centrifuged at 1,000 reads per million (rpm) for 1 hour with the addition of polybrene (5 ug/ml). Cells were transduced on 2 consecutive days with the shRNA retroviral constructs and then selected using puromycin (2 ug/ml).

### Retroviral constructs

Retroviral constructs to knockdown HNRNPL were generated by cloning oligos into the pSuper retroviral vector as previously described [25,52]. The following sequences targeting HNRNPL were used: HNRNPL A shRNA: GTTACAAAGACTTCAGTGA; HNRNPL B shRNA: GAACCATTACCAGATGAAA.

### Regenerated human epidermis

Human dermis obtained from the New York Firefighters Skin Bank was devitalized, cut, and placed upon an organotypic cassette. The bottom of the dermis was coated in matrigel, and the cassette was placed in keratinocyte growth medium, with the bottom of the dermis contacting the media and the top at the air interface. A total of 1 million keratinocytes were then seeded on the top of the dermis and allowed to regenerate and stratify for 4 days. The regenerated human skin were then harvested and embedded in OCT (Optimal Cutting Temperature) for sectioning and staining. Additional details for regenerating human skin have been previously described [25,36,52–54].

### Immunofluorescent staining

Sectioned tissue–derived regenerated human skin or normal adult human skin was fixed with 10% formalin solution (Sigma (St. Louis, MO) HT5012) for 12 minutes. Sections were blocked (PBS, 2% bovine serum albumin, 2.5% normal goat serum, and 0.3% Triton X-100) for 30 minutes. Primary antibodies were then added to blocking buffer and put onto sections for 1 hour. The following antibodies were used at the following concentrations: FLG at 1:200 (Abcam (Waltham, MA): ab3137), LOR at 1:400 (Abcam: ab198994), KI67 (Abcam: Ab16667) at 1:300, cleaved caspase-3 at 1:300 (Cell Signaling Technology (Danvers, MA): 9661S), HNRNPL (Bethyl (Montgomery, TX): A303-895A) at 1:400, K10 (Abcam: ab9025) at 1:300, and ITGB4 (Cell Signaling Technology: 14803S) at 1:150. Secondary antibodies were used at 1:500 for 30 minutes and included Alexa Fluor 555 goat anti-mouse IgG (Thermo Fisher Scientific: A21424) and Alexa Fluor 488 donkey anti-rabbit IgG (Thermo Fisher Scientific: A21206). Hoechst 33342 (Thermo Fisher Scientific: H3570) was used at 1:1,000 to stain nuclei.

### Hematoxylin–eosin staining

Sectioned tissue derived from regenerated human skin was fixed with 10% formalin solution (Sigma HT5012) for 12 minutes. Sections were then dipped in 0.25% Triton X-100 in PBS for 5 minutes. Hemotoxylin (Vector H-3401) staining was performed for 8 minutes, rinsed in water, and then dipped in acid alcohol (1% HCL in 70% ethanol). After subsequent rinsing, the sections were dipped in 0.2% ammonia water for 1 minute, rinsed again, and then dipped in 95% ethanol. Eosin (Richard-Allan Scientific (Kalamazoo, MI) 71304) staining was performed for 30 seconds followed by 95% ethanol rinsing for 1 minute. Sections were then put into 100% ethanol for 4 minutes followed by 2 minutes in Xylene.

### RNA extraction and analysis by RT-qPCR

The GeneJET RNA purification kit (Thermo Fisher Scientific: K0732) was used to extract RNA from cultured keratinocytes. RNA concentration for each sample was measured by nanodrop, and 1 μg of RNA was used for generating cDNA. This cDNA was generated using the Maxima cDNA Synthesis Kit (Thermo Fisher Scientific: K1642). qPCR was performed using this cDNA on the Bio-Rad (Hercules, CA) CFX96 real-time system. The housekeeping gene L32 was used for normalization of signal. Primer sequences for all genes tested are listed in S1 Data.

### Apoptosis assay

Four days after transfection, control and HNRNPL knockdown cells were stained with Annexin V conjugated to Alexa Fluor 488 (Life Technologies: A13201) and analyzed using the Guava flow cytometer (Millipore, Burlington, MA) according to manufacturer’s instructions and as previously described [17].

### Western blotting

IP samples or 20 to 80 ug of cell lysates were loaded into 4% to 12% Bis-Tris (Thermo Fisher Scientific: NW04122BOX) or 3% to 8% Tris-acetate (Thermo Fisher Scientific: EA03752BOX) and transferred to PVDF membranes. Membranes were blocked in 5% BSA in TBS. Membranes were exposed to primary antibodies in blocking buffer overnight at 4 degrees. The following primary antibodies were used: ITGB4 (Cell Signaling Technology: 14803S) at 1:200, ITGB1 (Cell Signaling Technology: 9699S) at 1:250, ITGA5 (Cell Signaling Technology: 4705S) at 1:200, ITGAV (Cell Signaling Technology: 4711S) at 1:250, HNRNPL (Bethyl: A303-895A) at 1:1,000, and RNA Pol II (Active Motif (Carlsbad, CA): 91151) at 1:1,000. The loading control Beta-Actin (Santa Cruz Biotechnology (Dallas, TX) sc-47778) was used at 1:5,000. The secondary antibodies used were donkey anti-rabbit IRDye 680RD (Li-Cor 926–68073) and donkey anti-mouse IRDye 800CW (Li-Cor 926–32212) at 1:5,000.

### RNA sequencing and bioinformatic analysis

Control or HNRNPLi cells were harvested 4 days after siRNA transfection. Independent biological duplicates were obtained for both CTLi and HNRNPLi, and total RNA was isolated using the GeneJET RNA purification kit (Thermo Fisher Scientific: K0732) and quantified by Nanodrop. RNA-seq was performed using the Illumina (San Diego, CA) NovaSeq 6000. RNA-seq libraries were prepared with NEBNext Ultra II Library Prep Kit (Illumina (San Diego, CA): E7760) then multiplexed, and approximately 40 million reads per sample were obtained. Pair-end reads were aligned to the GENCODE v19 transcriptome hg19 using TopHat2 with default settings [55]. Differential expression among samples was calculated using ANOVA from the Partek Genomic Suite (Partek, St. Louis, MO). Analysis of the read count distribution indicated that a threshold of 10 reads per gene generally separated expressed from unexpressed genes, so all genes with fewer than 10 reads were excluded from ANOVA analysis. Gene lists for significantly up-regulated or down-regulated genes were created using *p*-value < 0.05 and ≥2-fold change. Enriched GO terms for RNA-seq differentially expressed gene sets were identified using Enrichr [56,57]. Heatmaps for the RNA-seq data were generated using Partek Genomic Suite (http://www.partek.com/partek-genomics-suite/).

### ChIP sequencing and bioinformatic analysis

A total of 10 million cells and 5 μg of antibody were used for each antibody pulldown experiment for ChIP [17,27,54]. ChIP was performed using the following antibodies: HNRNPL (Bethyl: A303-895A), RNA Pol II (Active Motif: 91151), mouse IgG (Abcam: AB18413), and rabbit IgG (Millipore: 12–370). Cells for the RNA Pol II ChIP-qPCR or ChIP-Seq were fixed at a final concentration of 1% formaldehyde. Cells for the HNRNPL ChIP-qPCR or ChIP-Seq were fixed in both formaldehyde (1% final concentration, Thermo Fisher Scientific: 28908) and disuccinimidyl glutarate (2 mM final concentration, Thermo Fisher Scientific: 20593). qPCR results are represented as a percentage of input DNA. For ChIP-Seq, the ChIP DNA library was prepared using the TrueSeq DNA sample prep kit (Illumina). Sequencing was done on the HiSeq 4000 System (Illumina) using single 1 × 75 reads at the Institute for Genomic Medicine Core, UCSD. The ChIP-seq reads were processed by the ENCODE Transcription Factor and Histone ChIP-Seq processing pipeline (https://github.com/ENCODE-DCC/chip-seq-pipeline2) on our local workstation. The reads were first trimmed based on quality score before alignment to reference hg19; upon alignment and deduplication, the peak calling was then carried out by MACS2.2.4 with a cutoff q-value of 0.05 [58,59]. The heatmaps and metagene plots for the ChIP-Seq data were generated using ngs.plot [60]. Gene tracks were visualized using UCSC genome browser along with annotation tracks. The differential peaks between CTLi and HNRNPLi Pol II ChIP-Seq were determined using diffReps [61].

### Co-immunoprecipitation

Primary human keratinocytes were harvested in IP lysis buffer (25 mM Tris-HCl pH 7.4, 150 mM NaCl, 1 mM EDTA, 1% NP-40 and 5% glycerol) and sheared with a syringe. Moreover, 5 ug of HNRNPL (Bethyl: A303-895A) or control rabbit IgG (Millipore: 12–370) was conjugated to 50 ul of Protein G dynabeads (Life Technologies: 10004D) for 30 minutes at room temperature. Lysis buffer was diluted to 1 ml per sample and was added to the antibody conjugated beads and incubated overnight at 4 degrees. The following day, the beads were washed with IP lysis buffer and boiled in RIPA buffer (25 mM Tris-HCl (pH 7.6), 150 mM NaCl, 1% NP-40, 1% sodium deoxycholate, 0.1% SDS) supplemented with NuPAGE LDS Sample Buffer (Life Technologies: NP0008) to elute. Samples were then loaded for western blotting.

### Statistics

Statistical analyses were performed using GraphPad Prism 9. Histogram data are presented as the mean ± SD, and significant differences between 2 samples were determined by Student *t* tests. For groups of 3 or more, 1-way ANOVA with Tukey was used. Significant changes were defined as *p* ≤ 0.05.

## Supporting information

S1 Fig Knockdown of HNRNP genes shows that only HNRNPL is necessary for epidermal growth and blockade of premature differentiation. **(A)** Primary human keratinocytes were knocked down with control (CTLi), HNRNPFi, HNRNPH1i, or HNRNPH2i siRNAs, and the remaining mRNA levels of each gene was measured using RT-qPCR. qPCR results were normalized to *L32* levels. **(B)** CTLi, HNRNPFi, HNRNPH1i, or HNRNPH2i cells were seeded at 150,000 cells and counted 4 days later. Cell number was reported as a percentage of CTLi levels. **(C, D)** RT-qPCR for *IVL* (C) and *FLG* (D) mRNA expression in CTLi, HNRNPFi, HNRNPH1i, or HNRNPH2i cells after 4 days of knockdown. qPCR results were normalized to *L32* levels. **(E)** Primary human keratinocytes were knocked down with control (CTL shRNA) or HNRNPL (HNRNPL shRNA-A or shRNA-B) shRNAs delivered by retroviruses. HNRNPL shRNAs A and B are distinct shRNAs that target 2 different regions of the gene. Knockdown was measured using RT-qPCR. qPCR results were normalized to *L32* levels. **(F)** CTL shRNA, HNRNPL shRNA-A, and HNRNPL shRNA-B cells were seeded at 150,000 cells and counted 6 days later. Cell number was reported as a percentage of CTLi levels. **(G)** RT-qPCR for epidermal differentiation gene expression in CTL shRNA, HNRNPL shRNA-A, and HNRNPL shRNA-B cells after 6 days of knockdown. qPCR results were normalized to *L32* levels. *N* = 3 independent experiments for the entire figure. Mean values are shown with error bars = SD. *p < 0.05, ** ***p*** < 0.01, ***p < 0.001, ****p < 0.0001 (*t* test for A–D and 1-way ANOVA with Tukey for E–G). Primary data for this figure can be found in S1 Data. HNRNPL, heterogeneous nuclear ribonucleoprotein L; RT-qPCR, reverse transcription quantitative PCR; shRNA, short hairpin RNA; siRNA, small interfering RNA.
(EPS)

S2 Fig HNRNPL is expressed throughout the epidermis and is necessary to prevent apoptosis of the tissue. **(A)** Regenerated human skin using three-dimensional organotypic cultures made from CTLi or HNRNPLi cells were harvested after 4 days of culture. Immunostaining of apoptotic marker cleaved caspase 3 (green) and FLG (red) are shown. Nuclei are shown in blue (Hoechst staining). White arrows denote large areas of apoptotic cells. The dashed white lines denote the basement membrane zone. Scale bar = 40 μm. **(B)** Immunostaining of HNRNPL (green) and early differentiation marker K10 (red) on adult human skin. Nuclei are shown in blue (Hoechst staining). The dashed white lines denote the basement membrane zone. Scale bar = 40μm. **(C)** Western blot for HNRNPL in undifferentiated (day 0) and differentiated (days 3 and 5) primary human keratinocytes. Actin was used as a loading control. Representative images are shown. Primary data for this figure can be found in S1 Data. FLG, filaggrin; HNRNPL, heterogeneous nuclear ribonucleoprotein L; K10, keratin 10.
(EPS)

S3 Fig HNRNPL binds to regions enriched for skin transcription factors such as RUNX2 and SOX9. **(A)** De novo motif analysis of the 2,843 HNRNPL bound peaks using HOMER. **(B)** Gene track of *ITGB4* and its adjacent genes (*SAP30BP* and *UNK*). HNRNPL ChIP-Seq is shown in red. Y-axis shows RPM and red bar over gene tracks represent significant peaks. X-axis shows position along gene. **(C)** HNRNPL ChIP-qPCR on CTLi and HNRNPLi cells. IGG pulldowns in CTLi and HNRNPLi cells were used as a negative control. qPCR was used to determine the amount of binding to genes listed on the X-axis. Primers were targeted toward the TSS of each gene. Results are plotted as a percent of input. *N* = 3 independent experiments. Mean values are shown with error bars = SD. *p < 0.05, ** ***p*** < 0.01, ***p < 0.001, ****p < 0.0001 (*t* test was used and each sample compared to CTLi). Primary data for this figure can be found in S1 Data. ChIP-Seq, chromatin immunoprecipitation sequencing; HNRNPL, heterogeneous nuclear ribonucleoprotein L; IGG, immunoglobulin; RPM, reads per million; TSS, transcription start site.
(EPS)

S4 Fig HNRNPL binds and promotes the transcription of integrin/ECM genes through RNA Pol II. **(A)** Heatmap of Pol II ChIP-Seq in CTLi and HNRNPLi cells. Pol II ChIP-Seq signal is ranked by decreasing Pol II occupancy. X-axis shows −2 kb to +2 kb from the TSS. **(B)** Overlap of the HNRNPL bound genes that lost Pol II peaks with the down-regulated genes upon HNRNPL knockdown. **(C)** GO terms of the 111 overlapped genes from (B). **(D–F)** Gene tracks of *LAMA3* (D), *ITGA3* (E), and *FN1* (F). Pol II ChIP-Seq is shown in in CTLi (red) and HNRNPLi (green) cells. Y-axis shows RPM. Red or green bar over gene tracks represent significant peaks. X-axis shows position along gene. **(G)** RNA Pol II ChIP on CTLi and HNRNPLi cells. IGG pulldowns in CTLi and HNRNPLi cells were used as a negative control. qPCR was used to determine the amount of binding to genes listed on the X-axis. Primers were targeted toward the TSS of each gene. Results are plotted as a percent of input. *N* = 3. Mean values are shown with error bars = SD. *p < 0.05, ** ***p*** < 0.01, ***p < 0.001, ****p < 0.0001 (*t* test was used and each sample compared to CTLi). **(H)** Overlap of the HNRNPL bound genes (HNRNPL ChIP-Seq) with the genes that gain Pol II peaks upon HNRNPL depletion. **(I)** GO terms of the 430 overlapped genes from (H). Primary data for this figure can be found in S1 Data. ChIP-Seq, chromatin immunoprecipitation sequencing; ECM, extracellular matrix; GO, gene ontology; HNRNPL, heterogeneous nuclear ribonucleoprotein L; IGG, immunoglobulin; Pol II, polymerase II; RPM, reads per million; TSS, transcription start site.
(EPS)

S1 Table RNA-seq of HNRNPL knockdown primary human keratinocytes. Primary human keratinocytes were grown in proliferation conditions and knocked down for control (CTLi) or HNRNPL (HNRNPLi). Gene lists for significantly up-regulated or down-regulated genes were created using *p*-value < 0.05 and ≥2-fold change. HNRNPL, heterogeneous nuclear ribonucleoprotein L; RNA-seq, RNA sequencing.
(XLSX)

S2 Table ECM and hemidesmosome genes down-regulated upon HNRNPL depletion. List of the 69 ECM and hemidesmosome genes that are decreased in expression upon HNRNPL knockdown. ECM, extracellular matrix; HNRNPL, heterogeneous nuclear ribonucleoprotein L.
(XLSX)

S3 Table ChIP-Seq of HNRNPL in proliferating keratinocytes. List of the genes bound by HNRNPL including the coordinates where the peaks were found. ChIP-Seq, chromatin immunoprecipitation sequencing; HNRNPL, heterogeneous nuclear ribonucleoprotein L.
(XLSX)

S4 Table HNRNPL-bound genes that lost RNA Pol II peaks upon HNRNPL knockdown. List of the 495 genes bound by HNRNPL that lost RNA Pol II peaks upon HNRNPL depletion. HNRNPL, heterogeneous nuclear ribonucleoprotein L; Pol II, polymerase II.
(XLSX)

S1 Data Primary data for Figs 1–4 and S1–S4 Figs. (XLSX)

## Abbreviations

ChIP-Seq: chromatin immunoprecipitation sequencing
DMEM: Dulbecco’s Modified Eagle Medium
ECM: extracellular matrix
FLG: filaggrin
FN1: fibronectin1
GO: gene ontology
HNRNP: heterogeneous nuclear ribonucleoprotein
HNRNPK: heterogeneous nuclear ribonucleoprotein K
HNRNPL: heterogeneous nuclear ribonucleoprotein L
IP: immunoprecipitation
K10: keratin 10
lncRNA: long noncoding RNA
LOR: loricrin
MAPK: mitogen-activated protein kinase
Pol II: polymerase II
qPCR: quantitative PCR
RBP: RNA-binding protein
RNA-seq: RNA sequencing
rpm: reads per million
RT-qPCR: reverse transcription quantitative PCR
shRNA: short hairpin RNA
siRNA: small interfering RNA
TES: transcription end site
TNF: tumor necrosis factor
TSS: transcription start site
